# Supplementary material for: Identification by Reverse Vaccinology of Three Virulence Factors in Burkholderia cenocepacia That May Represent Ideal Vaccine Antigens
Source: Vaccines (Basel). 2023 May 30;11(6):1039. doi: 10.3390/vaccines11061039 (PMC10303873; doi:10.3390/vaccines11061039)
Supplement: Supplementary file 1 [file vaccines-11-01039-s001.zip › vaccines-2382392-supplementary.pdf]

Supplementary material

Table S1. Strains and plasmids used in this work.

***B. cenocepacia* strains**	**Properties**	**Reference**
B. cenocepacia K56-2	WT	[58]
ΔBCAL1524	K56-2 *ΔBCAL1524*	This work
ΔBCAM0949	K56-2 *ΔBCAM0949*	This work
ΔBCAS0335	K56-2 *ΔBCAS0335*	This work
K56-2 pAP20	K56-2 carrying pAP20	This work
ΔBCAL1524 pAP20	*ΔBCAL1524* carrying pAP20	This work
ΔBCAM0949 pAP20	*ΔBCAM0949* carrying pAP20	This work
ΔBCAS0335 pAP20	*ΔBCAS0335* carrying pAP20	This work
ΔBCAL1524 pAP20-*BCAL1524*	*ΔBCAL1524* carrying pAP20-*BCAL1524*	This work
ΔBCAM0949 pAP20-*BCAM0949*	*ΔBCAM0949* carrying pAP20-*BCAM0949*	This work
ΔBCAM0949 pAP20-*BCAM0949-BCAM0950*	*ΔBCAM0949* carrying pAP20-*BCAM0949-BCAM0950*	This work
ΔBCAS0335 pAP20-*BCAS0335*	*ΔBCAS0335* carrying pAP20-*BCAS0335*	This work
ΔBCAL1524 pSCrhaB2	*ΔBCAL1524* carrying pSCrhaB2	This work
ΔBCAM0949 pSCrhaB2	*ΔBCAM0949* carrying pSCrhaB2	This work
ΔBCAS0335 pSCrhaB2	*ΔBCAS0335* carrying pSCrhaB2	This work
ΔBCAL1524 pSCrhaB2-*BCAL1524*	*ΔBCAL1524* carrying pSCrhaB2-*BCAL1524*	This work
ΔBCAM0949 pSCrhaB2-*BCAM0949*	*ΔBCAM0949* carrying pSCrhaB2-*BCAM0949*	This work
ΔBCAM0949 pSCrhaB2-*BCAM0949-BCAM0950*	*ΔBCAM0949* carrying pSCrhaB2-*BCAM0949-BCAM0950*	This work
ΔBCAS0335 pSCrhaB2-*BCAS0335*	*ΔBCAS0335* carrying pSCrhaB2-*BCAS0335*	This work
***Escherichia coli* strains**	**Properties**	**Reference**
E. coli DH5α	*F^- Φ80lacZΔM15 Δ(lacZYA-argF) U169 recA1 endA1 hsdR17(rK^- mK^+) phoA supE44 thi-1 gyrA96 relA1λ^-.*	Laboratory stock
E. coli SY327	*araD Δ* (*lac pro*) *argE(Am) recA56 nalA λ pir,* Rif^r	M. Valvano
**Plasmids**		
pGPI-SceI-XCm	*ori R6K, ΩTpr, mob^+, containing the ISce-I restriction site,* Tp^r, Cat^r, *xylE*	[40]
pGPI-SceI-XCm -*ΔBCAL1524*	*pGPI-SceI-Xcm* carrying 5'- and 3'-flanking regions of *BCAL1524* for deletion	This work

pGPI-SceI-XCm −*ΔBCAM0949*	*pGPI-SceI-Xcm* carrying 5'- and 3'-flanking regions of *BCAM0949* for deletion	This work
pGPI-SceI-Xcm −*ΔBCAS0335*	*pGPI-SceI-Xcm* carrying 5'- and 3'-flanking regions of *BCAS0335* for deletion	This work
pDAI-SceI-SacB	pDA12 encoding the I*Sce*-I homing endonuclease, Tet^r, *sacB*	[40]
pRK2013	ori_{colE1}, RK2 *derivative,* mob^+, tra^+, Kan^r	[78]
pAP20	ori_{pBBR1} Cat^r, mob^+, P_{dhfr}	[41]
pAP20-*BCAL1524*	pAP20, *BCAL1524* under the control of P_{dhfr}	This work
pAP20-*BCAM0949*	pAP20, *BCAM0949* under the control of P_{dhfr}	This work
pAP20-*BCAS0335*	pAP20, *BCAS0335* under the control of P_{dhfr}	This work
pAP20-*BCAM0949-BCAM0950*	pAP20, *BCAM0949-BCAM0950* under the control of P_{dhfr}	This work
pSCrhaB2	ori_{pBBR1}, *rhaR*, *rhaS*, P_{rhaB}, Tp^r, mob^+	[42]
pSCrhaB2-*BCAL1524*	pSCrhaB2, *BCAL1524* under the control of P_{rhaB}	This work
pSCrhaB2-*BCAM0949*	pSCrhaB2, *BCAM0949* under the control of P_{rhaB}	This work
pSCrhaB2-*BCAS0335*	pSCrhaB2, *BCAS0335* under the control of P_{rhaB}	This work
pSCrhaB2-*BCAM0949-BCAM0950*	pSCrhaB2, *BCAM0949-BCAM0950* under the control of P_{rhaB}	This work

Rif^r, rifampicin resistance; Tp^r, trimethoprim resistance; Cat^r, chloramphenicol resistance; Tet^r, tetracycline resistance; Kan^r, kanamycin resistance.

Table S2. Primers used for deletion construction.

**Primer used for deletion construction**	
**Name**	Sequence (5'→ 3')
**ΔBCAL1524For1**	CAATATTGCATGCGGTACCCGTAGTGTACCGAACCGTACATTTCC
**ΔBCAL1524Rev2**	CTTGCCGTGCGTGCCGCCAGGGATAACCTTAATGTCCATTTCGTC
**ΔBCAL1524For2**	GACATTAAGGTTATCCCTGGCGGCACGCACGGCAAGTAATCCGAC
**ΔBCAL1524Rev2**	CAAGCTTCTTCTAGACTGGTCGCACCGACGACGTTGCCGAG
**ΔBCAM0949For1**	CAATATTGCATGCGGTACCGCAGCATCGCTATGCGCTGAACGAG
**ΔBCAM0949Rev1**	CACGCCCGCGAGCTTCAGGGAACGCATCGATTTGGCCATGCATGTTC
**ΔBCAM0949For2**	CCAAATCGATGCGTTCCCTGAAGCTCGCGGGCGTGTGATCGATG
**ΔBCAM0949Rev2**	CAAGCTTCTTCTAGAGGACTTGTCGACCGCACCGGCGTCG
**ΔBCAS0335For1**	CAATATTGCATGCGGTACCGTCCGAACTTTGAACGACGGGTGGC
**ΔBCAS0335Rev1**	GAACTGATAACCCGCACCCGAAATCTGCTTCCTCTTCATGGTCTTC
**ΔBCAS0335For2**	GAAGAGGAAGCAGATTTCGGGTGCGGGTTATCAGTTCTGATGCAGG
**ΔBCAS0335Rev2**	CCAAGCTTCTTCTAGACGGGCGTTTGAGGATCCAGGATTTTTC
**Primer for deletion check**	
**Name**	Sequence (5'→ 3')
**ΔBCAL1524checkFor**	CCTTGCTCACGATTTGATGCAAG
**ΔBCAL1524checkRev**	CAGCGCCTTGACCGTCGAGCTC
**ΔBCAM0949checkFor**	GCGTCGAGACGTCGCTCGCGTATGCGTG

**ΔBCAM0949checkRev**	GAAGAACGGCTGGCTCCAGTCGCCGAGC
**ΔBCAS0335checkFor**	CGTTTGCGGAATGTGTGTATTTTGCG
**ΔBCAS0335checkRev**	GAACTGCCGAGTCTCGATGCCGTGCA

Table S3. Primers used for complementation.

**Primer used for complementation**	
**Name**	Sequence (5’→ 3’)
**pSCrhaB2BCAL1524for**	GAAATTCAGCAGGATCACATATGGACATTAAGGTTATCCCTCATGG
**pSCrhaB2BCAL1524rev**	CTGCAGGTCGACTCTAGAGTTACTTGCCGTGCGTGCCGCC
**pSCrhaB2BCAM0949for**	GAAATTCAGCAGGATCACATATGGCCAAATCGATGCGTTCC
**pSCrhaB2BCAM0949rev**	CTGCAGGTCGACTCTAGAGTCACACGCCCGCGAGCTTCAG
**pSCrhaB2BCAM0949BCAM0950rev**	CTGCAGGTCGACTCTAGAGTCAATGCGCGCTGCCCGCGC
**pSCrhaB2BCAS0335for**	GAAATTCAGCAGGATCACATATGAAGAGGAAGCAGATTTCGG
**pSCrhaB2BCAS0335rev**	CTGCAGGTCGACTCTAGAGTCAGAACTGATAACCCGCACCG
**pAP20BCAL1524for**	GATTCACAAGAAGGATTCGGATGGACATTAAGGTTATCCCTC
**pAP20BCAL1524rev**	CTTGCATGCCTGCAGGTCGACTTTACTTGCCGTGCGTGCCGCCG
**pAP20BCAM0949for**	GATTCACAAGAAGGATTCGGATGGCCAAATCGATGCGTTCC
**pAP20BCAM0949rev**	CTTGCATGCCTGCAGGTCGACTTCACACGCCCGCGAGCTTCAG
**pAP20BCAM0949BCAM0950rev**	GCATGCCTGCAGGTCGACTTCAATGCGCGCTGCCCGCGCC
**pAP20BCAS0335for**	GATTCACAAGAAGGATTCGGATGAAGAGGAAGCAGATTTCGG
**pAP20BCAS0335rev**	CTTGCATGCCTGCAGGTCGACTTCAGAACTGATAACCCGCACCG

Table S4. Antimicrobial susceptibilities (µg/ml) of *Burkholderia cenocepacia* wt, mutants and of the complemented strains.

	ANTIBIOTICS									
STRAIN	AMK	AZT	CIP	LVX	MEM	MIN	NAL	PIP	SPX	TOB
**K56-2**	≥256	≥256	2	4	8	8	16	128	4	≥256
ΔBCAL1524	≥256	256	2	4	8	4	8	128	4	≥256
ΔBCAM0949	≥256	256	2	4	4	16	16	**32**	4	≥256
ΔBCAS0335	≥256	256	4	4	8	**64**	16	128	4	≥256
**K56-2 pSCrhaB2**	≥256	≥256	4	8	16	4	16	128	4	≥256
ΔBCAL1524 **pSCrhaB2**	≥256	≥256	≤2	4	8	≤2	8	128	4	≥256
ΔBCAL1524 **pSCrhaB2*BCAM1524***	≥256	≥256	≤2	4	16	≤2	16	64	4	≥256
ΔBCAM0949 **pSCrhaB2**	≥256	≥256	≤2	4	8	≤2	16	**16**	4	≥256
ΔBCAM0949 **pSCrhaB2*BCAM0949***	≥256	≥256	≤2	4	4	≤2	4	**128**	4	≥256
ΔBCAS0335 **pSCrhaB2**	≥256	256	≤2	4	4	32	16	64	2	≥256
ΔBCAS0335 **pSCrhaB2*BCAS0335***	≥256	≥256	≤2	4	8	**4**	8	128	4	≥256

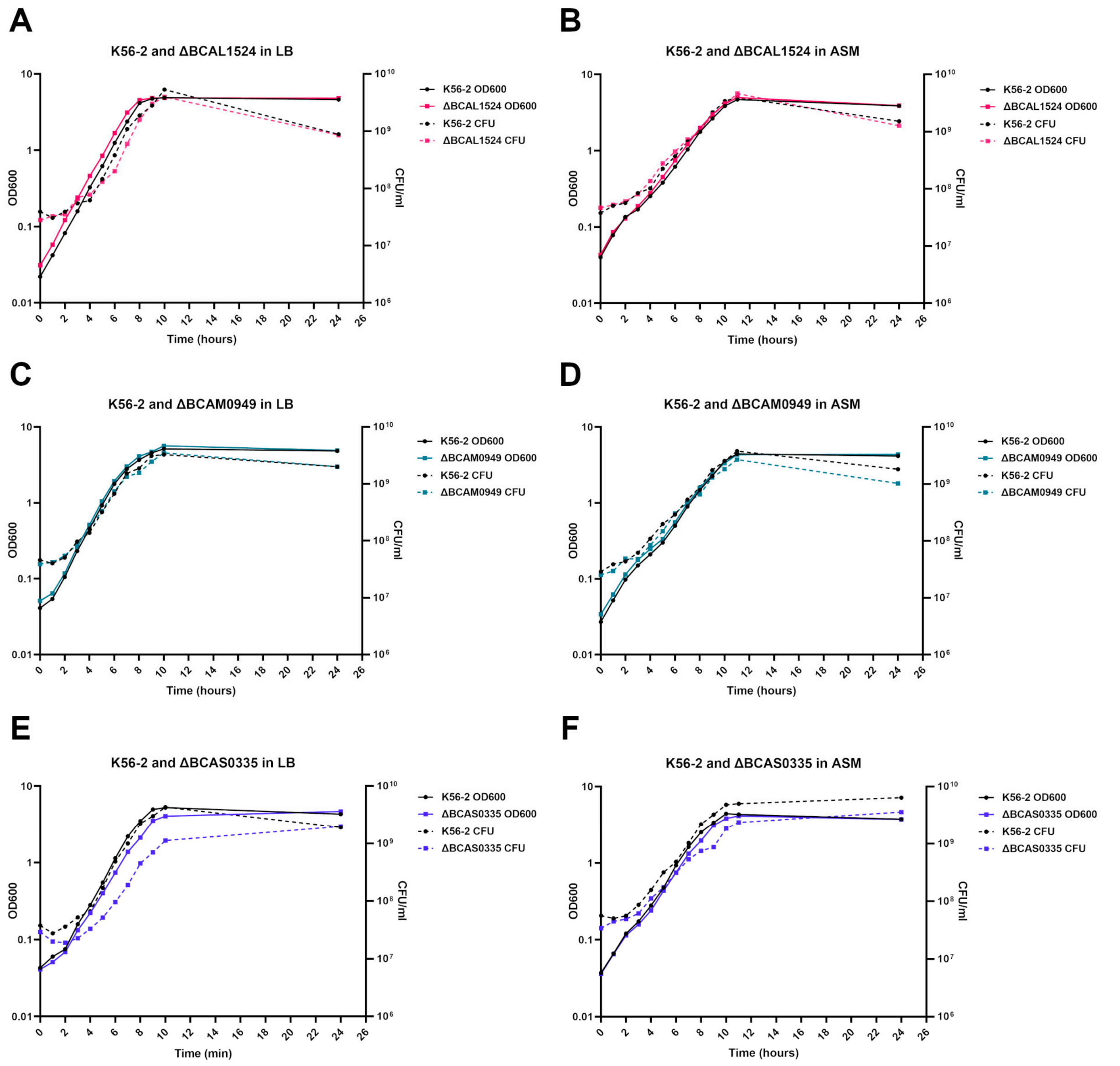

Figure S1. Growth curves of WT and deleted strains. OD_{600} absorbance and CFU of WT and ΔBCAL1524 in LB (A) and ASM (B). OD_{600} absorbance and CFU of WT and ΔBCAM0949 in LB (C) and ASM (D). OD_{600} absorbance and CFU of WT and ΔBCAS0335 in LB (E) and ASM (F).

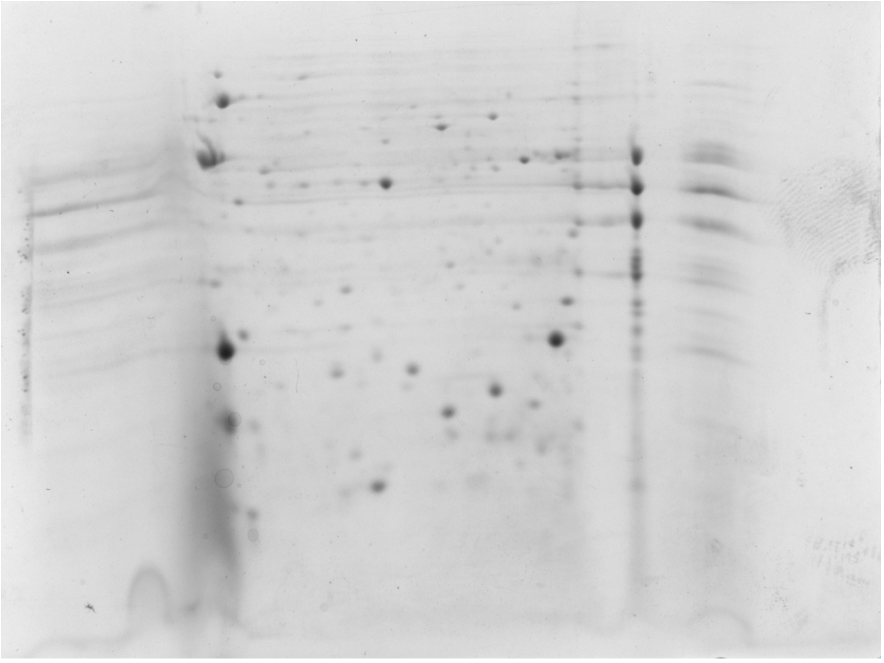

Figure S2. Two-dimensional gel electrophoresis (2DE)-stained gel with Coomassie of K56-2 OMV.

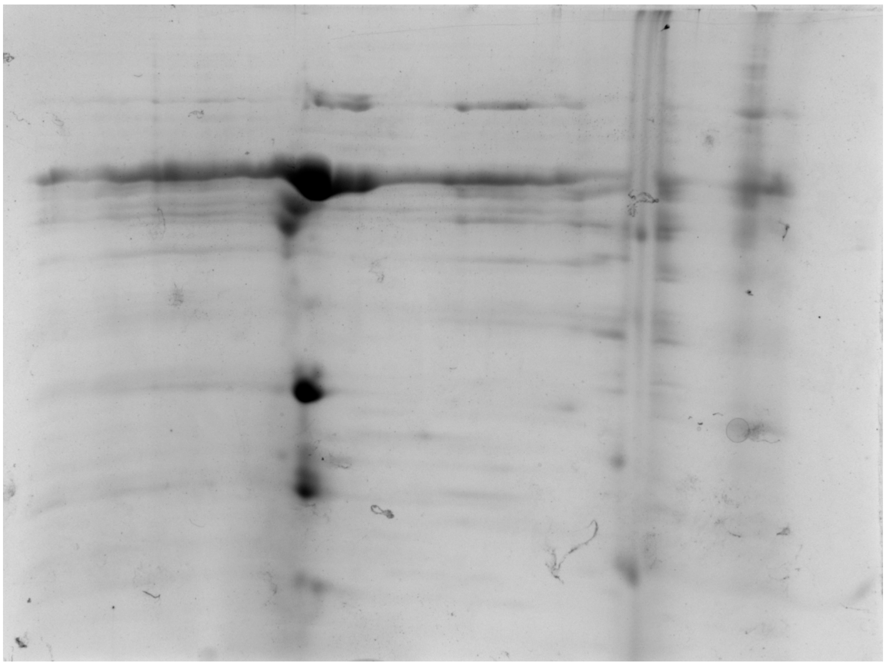

Figure S3. Two-dimensional gel electrophoresis (2DE)-stained gel with Coomassie of ΔBCAL1524 OMV.

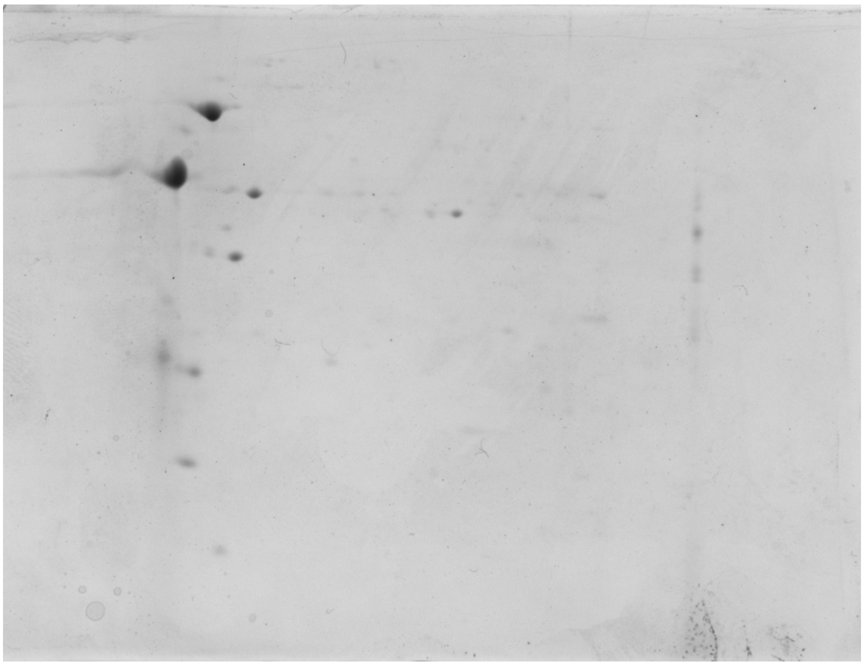

Figure S4. Two-dimensional gel electrophoresis (2DE)-stained gel with Coomassie of ΔBCAM0949 OMV.

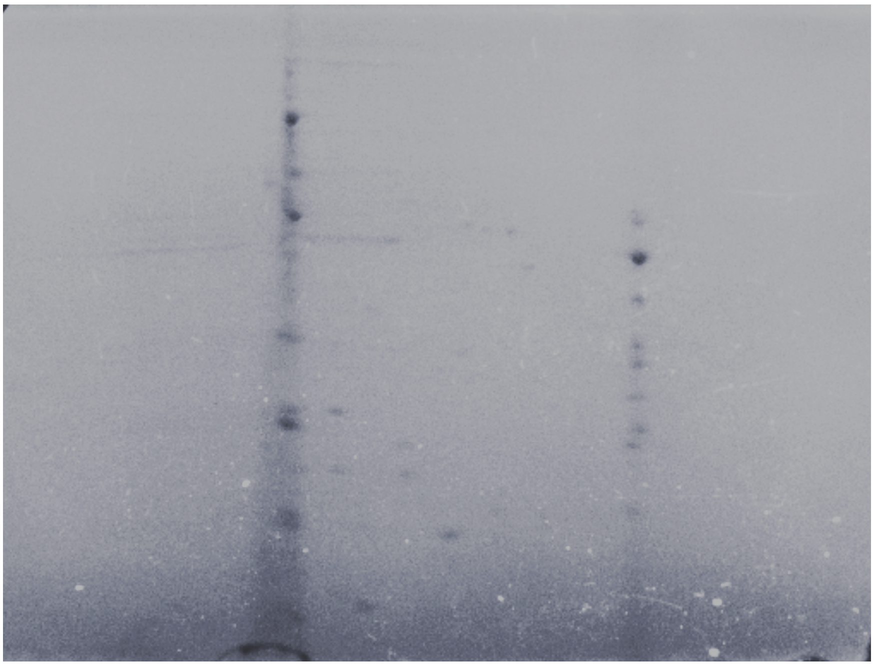

Figure S5. Two-dimensional gel electrophoresis (2DE)-stained gel with Coomassie of ΔBCAS0335 OMV.

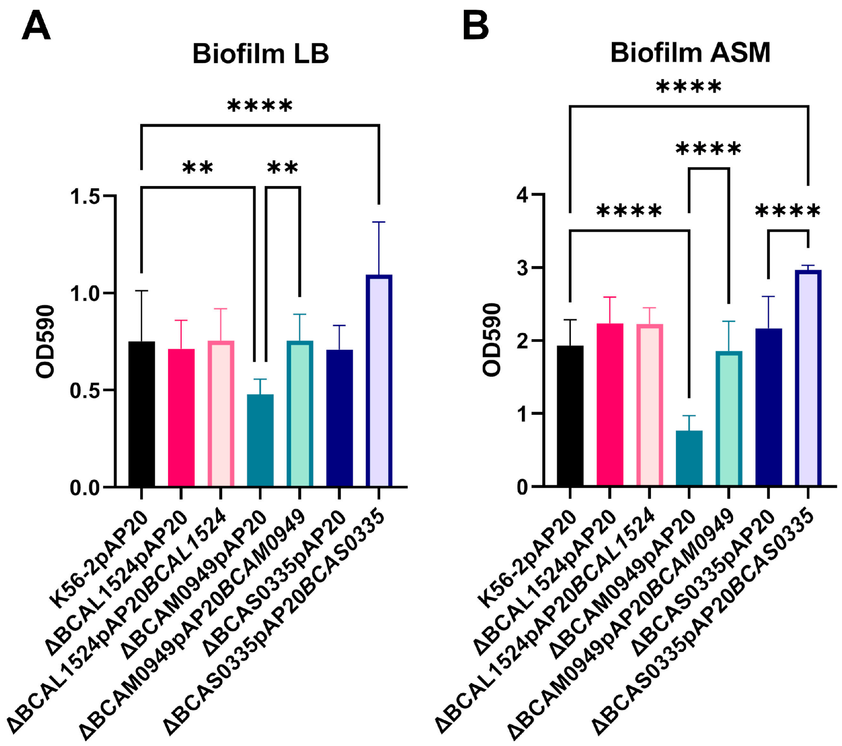

Figure S6. Graphical representation of the OD_{590} measured after the crystal violet assay comparing the biofilm formation of *B. cenocepacia* complemented strains. (A) Biofilm of complemented strains in LB and (B) in ASM. (** $p< 0.01$, **** $p< 0.0001$ one-way ANOVA test).

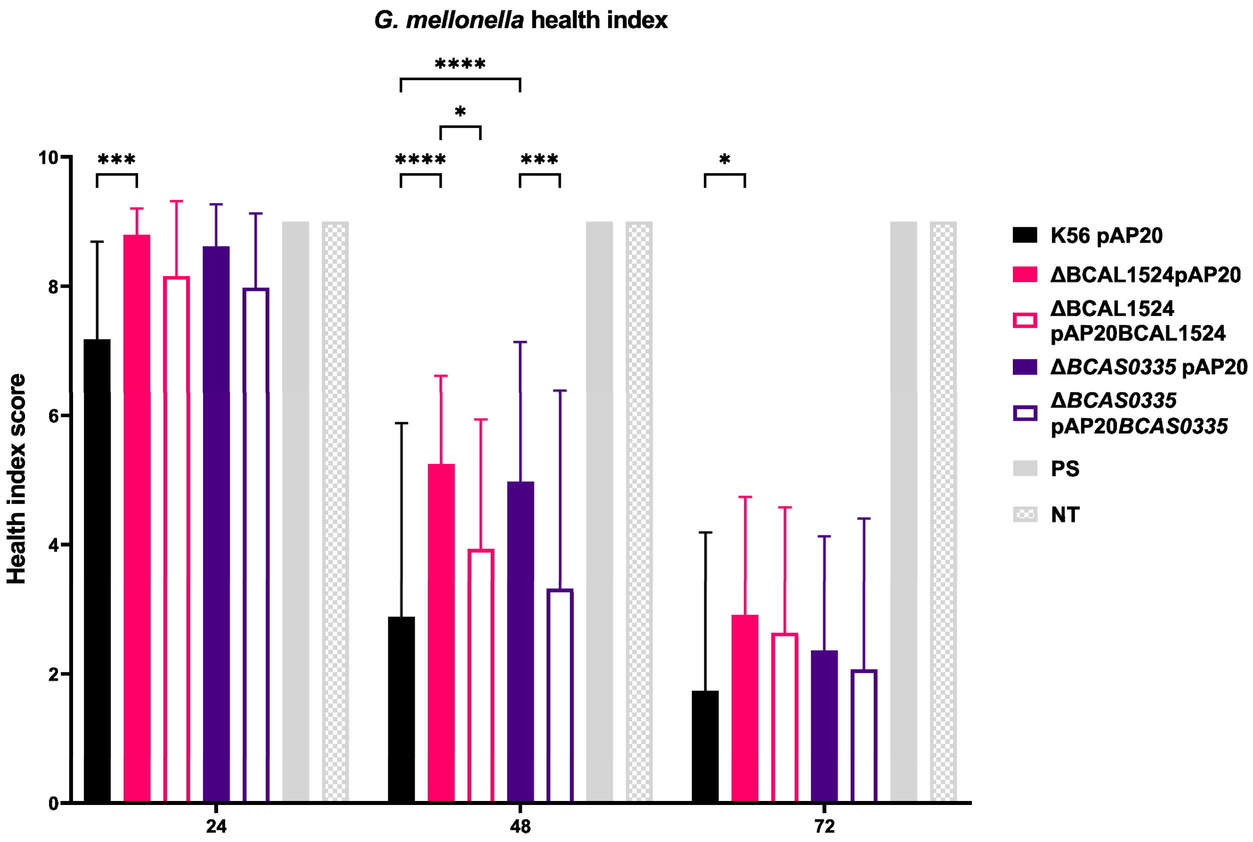

Figure S7. Graphical representation of the *G. mellonella* health index score. *G. mellonella* moths were injected with the complemented strains or with physiological solution or not injected (*p< 0.1, **p< 0.01, ***p<0.001, **** p< 0.0001 two-way ANOVA test).
